# What to target? Interventions to modulate key mechanisms underlying the trajectories of affective disorders in the transregional Collaborative Research Center 393

**DOI:** 10.1007/s00115-025-01929-0

**Published:** 2025-11-27

**Authors:** Elisabeth J. Leehr, Joachim Groß, Stefan G. Hofmann, Philipp Kanske, Tilo Kircher, Igor Nenadić, Winfried Rief, Philipp Ritter, Allan Young, Katharina Förster

**Affiliations:** 1 Institute for Translational Psychiatry, University of Münster, Albert-Schweitzer-Campus 1, Building A9, 48149 Münster, Germany; 2 Institute for Biomagnetism and Biosignal Analyses, University of Münster, Münster, Germany; 3 Department of Psychology, Philipps-Universität Marburg, Marburg, Germany; 4 Institute of Clinical Psychology and Psychotherapy, TUD Dresden University of Technology, Dresden, Germany; 5 Department of Psychology, Faculty of Psychology and Educational Sciences, Babeș-Bolyai University, Cluj-Napoca, Romania; 6 Department of Psychiatry and Psychotherapy, Universität Marburg, Marburg, Germany; 7 Department of Psychiatry and Psychotherapy, Technische Universität Dresden, Dresden, Germany; 8 Child and Adolescent Psychotherapy, Institute of Psychology, Faculty of Psychology and Movement Science, Universität Hamburg, Hamburg, Germany

**Keywords:** Mood disorders, Affective disorders, emotion regulation, Expectation violation, Empathy, Circadian rhythm, Stimmungsstörungen, Affektive Störungen, Emotionsregulation, Erwartungsverletzung, Empathie, Zirkadianer Rhythmus

## Abstract

**Background:**

Affective disorders are associated with an enormous disease burden, necessitating research on the mechanisms of effective treatments.

**Objectives:**

This article introduces the intervention projects of the transregional Collaborative Research Center 393 (CRC/TRR 393). By employing targeted interventions, we aim to induce modifiability in the key mechanisms underlying the trajectories of affective disorders studied in CRC/TRR 393: emotion regulation, expectation, social cognition, and (circadian) cognitive–behavioral rhythms.

**Materials and methods:**

The longitudinal design of the CRC/TRR 393 study will examine four interventions targeting specific mechanisms in subgroups of the German Mental Health Cohort (GEMCO). GEMCO includes patients with a current or lifetime diagnosis of major depressive disorder (MDD), bipolar disorder (BD), and healthy control (HC) participants. Multilevel measurements of these targeted mechanisms will allow us to investigate their fine-grained modifiability and their effects on disease trajectories.

**Results:**

The results will provide insights into how these mechanisms can be modified. Together with the CRC/TRR 393 mechanisms projects, we will examine the effects of key cognitive–emotional mechanisms on disease trajectories.

**Conclusion:**

For the first time, the modifiability of four key mechanisms underlying the trajectories of affective disorders will be investigated both cross-sectionally and longitudinally.

**Supplementary Information:**

The online version of this article (10.1007/s00115-025-01929-0) contains an additional table.

## Abstract (de)

**Hintergrund:**

Affektive Störungen gehen mit einer erheblichen Krankheitslast einher. Daraus ergibt sich ein dringender Forschungsbedarf hinsichtlich der Mechanismen wirksamer Behandlungsansätze.

**Ziel der Arbeit:**

Im vorliegenden Artikel werden die Interventionsprojekte des Sonderforschungsbereichs/Transregio (SFB/TRR) 393 übersichtlich zusammengefasst. Wir streben an, durch gezielte Interventionen Veränderungen in spezifischen Schlüsselmechanismen, die dem Verlaufen affektiver Störungen zugrunde liegen, herbeizuführen. Im Fokus stehen dabei folgende 4 Mechanismen: *Emotionsregulation, Erwartungen, soziale Kognition *sowie *(zirkadiane) kognitiv-behaviorale *Rhythmen.

**Material und Methoden:**

Im Kontext der SFB/TRR-393-Teilprojekte werden 4 interventionsbasierte Ansätze im Längsschnitt untersucht, die jeweils auf einen der genannten Mechanismen fokussieren. Die Studien werden in Subgruppen der German Mental Health Cohort (GEMCO) durchgeführt. Die GEMCO-Stichprobe setzt sich aus Patient:innen mit akuter oder remittierter depressiver Störung (MDD), bipolarer Störung (BP) und Individuen ohne psychische Erkrankung zusammen. Mittels multimodaler Erhebungen wird die Modifizierbarkeit der angesprochenen Mechanismen sowie deren Effekte auf die Verläufe affektiver Störungen analysiert.

**Ergebnisse:**

Wir erwarten, Erkenntnisse darüber zu erhalten, wie die jeweiligen Mechanismen durch Interventionen gezielt verändert werden können. In enger Verknüpfung mit den assoziierten Mechanismusprojekten des SFB/TRR 393 sollen darüber hinaus deren Auswirkungen auf die Krankheitsverläufe umfassend untersucht werden.

**Schlussfolgerung:**

Erstmals wird die Veränderbarkeit von 4 zentralen kognitiv-emotionalen Schlüsselmechanismen, die maßgeblich an den Verläufen affektiver Störungen beteiligt sind, systematisch sowohl im Querschnitt als auch im Längsschnitt untersucht.

## Introduction

According to German S3 guidelines on the treatment of unipolar and bipolar disorders, first-line treatment for affective disorders comprises psychotherapy and/or psychopharmacological treatment for unipolar depression, depending on the severity [1, 2], and psychopharmacological treatment combined with psychotherapy for bipolar disorder (BD; [2]). However, only up to one third (20–30%) of patients affected by major depressive disorder (MDD) in Germany are treated with psychotherapy [3], and only about half of these patients receive adequate treatment [4]. Additionally, more than 50% of patients with MDD do not respond to evidence-based psychotherapeutic treatment [5], and most treatment studies only have up to 1‑year follow-up assessments and are thus not suitable for investigating the long-term impact of treatment on disease trajectories.

Trajectories of affective disorders are dynamic, with recurrence reported in more than 50% of cases of MDD within 5 years [6] and up to 60% recurrence within 1 year following an initial (hypo-)manic episode in BD [7, 8]. Investigating the key mechanisms associated with these symptom changes holds great promise for improving our understanding of the course and maintenance of affective disorders (see [9]).

The transregional Collaborative Research Center 393 (CRC/TRR 393) study aims to investigate comprehensively the trajectories of affective disorders in a sample comprising patients with current or lifetime MDD, patients with BD, and healthy control (HC) participants, collectively known as the German Mental Health Cohort (GEMCO; for more details, see [10–12]).

Within this framework we investigate the effect of interventions targeting key mechanisms underlying the trajectories of affective disorders [9, 13]: emotion regulation (affective), expectation (cognitive), social cognition (behavioral), and circadian rhythms (physical). The four key mechanisms are not separate from each other but are interconnected and together contribute to symptom fluctuations, recurrences, remissions, and overall disease course. They have all been studied largely in isolation, but there is evidence of interactions between them (e.g., [14–16]), underscoring the importance of integrated investigation.

We take advantage of the longitudinal design and the tandem mechanistic projects [9] focused on specific mechanisms and their modifiability. Together with the interdisciplinary group of researchers involved in CRC 393, this enables a multilevel investigation [10]. Studying the effects of modifiability in these mechanisms on disease trajectories provides the opportunity to draw much stronger, even causal claims about their role in affective disorders. Finally, finding ways to modulate the four targeted key mechanisms will pave the way for the evidence-based development of innovative treatment options. In this funding period, we aim to investigate each intervention with a focus on the modifiability of the targeted key mechanisms. However, the joint research program previously described by Kircher et al. [10] will already enable us to delineate specific hypotheses regarding their interconnection and provide preliminary insights into options for personalized treatment selection. For follow-up research, applying adaptive trial designs offers a way to optimize the set of interventions within a modular treatment approach.

Here, we provide a comprehensive overview of the intervention projects (see also Table e1 in the online supplement) that are conducted within the framework of CRC/TRR 393.

## Cognitive reappraisal-based emotion regulation training (project C01)

Emotion regulation has been extensively investigated in the context of affective disorders (for a review, see [17]). Research indicates that individuals with depression, in particular, demonstrate a narrow repertoire of emotion regulation strategies and encounter difficulties in selecting strategies that are appropriate to the individual context [18]. One of the most widely investigated (and health-promoting) emotion regulation strategies is cognitive reappraisal (CR). This strategy involves reframing one’s thoughts in emotional situations [19] and can modulate emotional responses through goal-dependent evaluations of the situation or the stimulus. Cognitive reappraisal strategies include psychological distancing (e.g., “From an outside perspective, everything looks organized, and in a few weeks, this will feel manageable”) and reinterpretation (“This is one of the best hospitals; the operation will likely go well”), both of which reduce negative affect [20].

In our proof-of-concept study focusing on CR-based emotion regulation training (CR-ERT) in patients with current or remitted depression from GEMCO, patients will be randomized to either an 8‑week CR-ERT or an active control condition. The impact of the CR-ERT on ER flexibility will be assessed in two ways: firstly, behaviorally (via intensive ecological momentary assessment); and secondly, neurobiologically, using pre- and post-intervention multimodal assessments as described in the related mechanism project (see [9]). The role of childhood maltreatment as a risk factor for ER difficulties will also be examined. The study protocol and outcomes have been preregistered in the German Clinical Trials Register (DRKS00036315).

The CR-ERT will be delivered as a digital training program via a smartphone application. Participants will receive weekly 10-min videos introducing a CR technique, along with a daily workbook for practice. The CR techniques comprise the following:

- *Probability overestimation:* identifying and testing distressing thoughts for exaggerated probabilities
- *Catastrophic thinking:* exploring catastrophic thoughts by mentally following them to their logical conclusion
- *Perspective taking:* practicing third-person observation of distressing situations
- *Distancing:* learning that thoughts are not facts, using metaphors (e.g., a stable mountain vs. a storm) to cultivate detached mindfulness

The active control group will complete cognitive exercises targeting attention, verbal and executive functioning, and spatial perception, accompanied by instructional videos and daily workbook tasks.

We hypothesize that CR-ERT, compared to the control condition, will enhance ER flexibility by broadening the strategy repertoire and improving context-sensitive and adequate strategy selection. These behavioral improvements are expected to be reflected in neural changes in the underlying neural emotion-processing systems. Primary outcomes comprise several data levels, such as changes in ER flexibility (ER choice sensitivity and variability from baseline to follow-up) derived from the intense sampling measures of ERT compared to the control group (also: passive control). Additionally, magnetic resonance imaging (MRI) is used to assess structural plasticity (frontolimbic brain regions) and functional brain plasticity in ER and emotion perception networks between baseline and follow-up in C01, including structural and functional connectivity measures, derived from the ER choice and emotion perception paradigm (B01, [9]) and the ER paradigm (S02, [21]) for the GEMCO cohort. We further aim to explore whether targeting ER flexibility will also improve clinical outcomes (change in the number and frequency of inflection signals/depressive episodes) of participating patients with affective disorders.

## Expectation violation and reward sensitivity as targets in psychological interventions (project C02)

Expectation plays a crucial role in the development and maintenance of affective disorders [22]. In acute depression, individuals often hold negative expectations and beliefs about themselves and the future. In depression, reduced reward sensitivity often prevents positive experiences from updating negative expectations [23]. Only few interventions directly address expectancy violation and reward sensitivity. Patients with depression show blunted psychological and neural responses to rewards, such as monetary gains in gaming tasks [24]. In a randomized clinical trial focusing on reward sensitivity, short-term and long-term analyses demonstrated that improvements in reward sensitivity were most accurately predicted by changes in depressive expectations [25]. Therefore, we hypothesize that an approach focusing on expectation violation will be more effective than an intervention that exclusively focuses on reward sensitivity. Importantly, expectancy violations alone may not automatically lead to expectation change—reward sensitivity can modulate whether expectancy violations alter beliefs.

In the present study, 150 patients with acute MDD from GEMCO and associated outpatient clinics will be randomized into three arms: expectation-focused treatment, reward sensitivity-focused treatment (active control), and a waiting list control. The treatment will be administered by psychotherapists in training in face-to-face group settings over a period of 5 weeks, comprising 10 sessions, with two sessions held per a week. Assessments will be conducted at baseline, post-treatment, and at 6‑ and 12-month follow-ups. The primary objective of these assessments is to measure changes in expectation- and reward-related processes at behavioral and neural levels.

In the *expectation-focused* treatment arm, patients explore their dysfunctional expectations and their role in maintaining depressed mood, and they specify them for testable predictions. Based on this, patients engage in behavioral experiments designed to challenge these expectations. Participants are instructed to maintain daily diaries, integrating experiences from everyday life into therapy (see [26]).

In the *reward sensitivity-focused* treatment arm, patients are introduced to the concept of reward sensitivity and its role in depression. Positive events will be triggered to enhance the psychological effects of positive experiences, thereby also including some strategies from mindfulness-based treatments. The focus of the program will be on (a) increasing sensitivity for the detection of reinforcing events, (b) attention-focusing and perception amplification strategies for positive events, and (c) cognitive appraisal strategies for positive events (adapted version from [27]). As in the expectation-focused arm, diaries will be used to deliver the material to work with in therapy sessions.

In the *waiting list arm,* participants will either wait for treatment or continue any ongoing treatments, but they will not receive additional depression-specific interventions during the study period. Sample sizes and effect sizes are based on earlier mechanism-centered interventions focusing on reward sensitivity and on behavioral activation programs (e.g., [25]), with *d* = 0.3 as a minimum effect size of interest. The primary outcome measures comprise changes in Hamilton Rating Sclae for Depression (HAMD) [28] and MRI-based parameters derived from mechanism-specific paradigms (fMRI), with insula region of interest (ROI) and dorsolateral prefrontal cortex (dlPFC) changes in task-based activation post- vs. pre-treatment. Further, we anticipate both active treatments to outperform the waiting list approach in reducing depressive symptoms. The expectation-focused intervention is hypothesized to be more effective than the reward-sensitivity intervention. Additionally, different neural changes are expected in the active treatment groups, reflecting their targeted mechanisms.

## Positive social affect training (project C03)

Humans are inherently social beings, and social distress plays a significant role in the development and maintenance of affective disorders [29]. Research suggests that social stress transmission—defined as the process by which another person’s stress affects one’s own well-being (i.e., empathic distress)—may be a key mechanism linking social distress to depression [30]. Building on this concept, we will evaluate the effectiveness of *positive social affect training* (PSAT) in patients with depression and their intimate partners, compared to a mindfulness-based active control. The PSAT targets coping with a partner’s stress and alleviating empathic stress. The impact of partners’ recent stressful events, which may cause empathic distress in patients, will also be examined. A total of 100 patients with MDD who are currently euthymic and 100 HC participants from the GEMCO cohort and their partners (*n* = 400 in total) will participate in the study. Half of the dyads will receive an 8‑week PSAT, while the other half will engage in a matched, non-social mindfulness-based intervention. Previous studies that used similar social interaction tasks in healthy individuals showed effects of approximately *d* = 0.3 on behavioral data of empathic affect and affective capacities [31]. The study protocol and outcomes have been preregistered in the German Clinical Trials Register (DRKS00037543).

Both intervention conditions will take place for 8 weeks and will mainly be provided via an app. However, an open session for questions will be held weekly, alongside mandatory online meetings with the trained researcher at baseline and after 4 and 8 weeks of intervention. During the whole intervention, training performance will be assessed via ecological momentary assessment (EMA).

The PSAT targets the reduction in negative empathic affect transmission between partners through two daily exercises that are practiced by the dyads together. The intervention consists of two highly structured mental training exercises: *compassion-focused meditation* (core exercise 1) and *emotion-focused dialogue* (core exercise 2). The exercises last 10 min each and are practiced a minimum of 5 days per week during the intervention period.

The *meditation* fosters positive, caring emotions toward the self and others. Partners engage in meditation in unison, with a focus on themselves, guided by online instructions and audio scripts. This practice has been shown to enhance interpersonal connectedness and to reduce depressive symptoms [32].

The *emotion-focused dialogue* aims to improve empathy and compassion in face-to-face communication. Partners are seated together (or a video call is used if they are apart) and each of them speaks for 2.5 min about a difficult emotional event from the past 24 h, followed by 2.5 min discussing an event from the past 24 h for which they feel grateful. The listener is instructed to maintain compassionate attention without interrupting. Then, the roles are reversed.

This dialogue fosters nonjudgmental acceptance and mutual understanding [33].

The structurally equivalent control condition is a non-social mindfulness intervention, practiced individually without partner involvement, which consists of two core exercises of 10 min duration each: *mindful body scan* and *mindful breathing*. These exercises are widely used in meditation training programs such as mindfulness-based stress reduction (MBSR). This type of mindfulness-based meditation has been confirmed to have specific effects on well-being but not on compassion or related constructs [31]. During the *body scan*, participants systematically direct their attention to different body parts, enhancing interoceptive awareness (for more information, see [34]).

In the *mindful breathing exercise*, participants are instructed to deliberately concentrate on their breathing, without attempting to control it. It has been proven to train participants to intentionally focus attention, maintain stable attention toward a specific focus, and redirect attention when distraction or mind-wandering occurs [35].

We hypothesize that, compared to the mindfulness control, the PSAT will improve social interaction quality, reduce empathic distress, and enhance socio-affective capacities on neural, behavioral, and biological levels [36, 37]. Thus, the primary outcome will be changes in self-reported empathy assessed during the EmpaTom—an experimental task to assess empathy. Additionally, we expect long-term reductions in depressive symptoms, supporting the PSAT as a preventive and therapeutic strategy in depression [38]. Effects on (subclinical) depressive symptoms will be assessed over a 24-month follow-up.

## Circadian rhythms—a randomized dose–response study of wake therapy (project C04)

There is a strong, bidirectional relationship between symptom changes and alterations in cognitive–behavioral circadian rhythms in patients with affective disorders (e.g., sleep). Chronotherapeutic treatments—such as wake therapy—have demonstrated therapeutic efficacy in treating episodes of depression in MDD and BD, despite uncertainties regarding the precise causal mechanism of symptom change [39]. Current models suggest that disrupted neuroplasticity in the left dlPFC during episodes of depression may be a relevant contributing factor. We aim to refine this model by investigating the specific role of rapid eye movement (REM) sleep in relation to mood and neuroplasticity. To this end, we will study 63 patients with unipolar and bipolar depression, currently depressed, using electroencephalography (EEG) and transcranial magnetic stimulation-EEG (TMS-EEG), applying partial wake therapy as a model for inducing rapid and controlled symptom changes. A randomized dose–response protocol will assess the effects of curtailed sleep, aiming to reduce varying amounts of REM sleep, on synaptic density in critical cortical regions and relate this to symptom change.

The indices of neuroplasticity will be derived from TMS-EEG responses and the non-periodic (1/f) component of resting-state EEG.

This 4‑day intervention will investigate neuroplasticity, REM sleep regulation, and antidepressant response to sleep curtailment under tightly controlled circadian and behavioral conditions. The design is informed by robust evidence that REM disinhibition (shortened REM latency, increased REM density) and potentially altered synaptic downscaling during sleep are trait markers of affective disorders and may predict clinical trajectories [40].

Participants initially screened as outpatients will be admitted during the intervention to ensure protocol adherence. The intervention will be administered by trained researchers. The primary objective of this study is to reduce varying amounts of REM sleep and evaluate the impact on density in critical brain regions such as the dlPFC as quantified with EEG and TMS-EEG.

Chronotype will be assessed using the ultra-short version of the Munich ChronoType Questionnaire (μMCTQ). Participants will begin a controlled schedule with fixed wake/sleep times and scheduled light exposure. Resting-state EEG (2 h post-wake and nocturnal recordings) and TMS-EEG will be recorded, alongside measurements of brain-derived neurotrophic factor (BDNF) and assessments with the Montgomery–Åsberg Depression Rating Scale (MADRS), which will be conducted several times. Participants will be blinded to group allocation until wake time. During enforced wakefulness (dim light, 10 lx), quiet activities will be permitted, and adherence will be monitored.

Based on previous sleep deprivation studies, the design has sufficient power (*N* = 63, 80% power, α = 0.05) to detect a medium effect size (*f*^2^ ≈ 0.23) linking REM curtailment to neuroplasticity changes. The primary outcome is the modulation of synaptic plasticity in the left dlPFC, which is quantified using TMS-evoked EEG responses. This is complemented by resting-state EEG aperiodic slope and theta power, which are indices of excitation–inhibition balance and synaptic strength, respectively. Secondary outcomes include detailed REM sleep architecture (latency, density, %REM), serum levels of mature BDNF and proBDNF as molecular markers of plasticity, and acute mood changes, as measured with the MADRS. We hypothesize that baseline REM disinhibition and reduced EEG aperiodic slope during REM reflect trait vulnerability, as previously observed in both MDD and BD. We further expect that the extent of overnight synaptic downscaling, as indicated by steeper post-intervention EEG aperiodic slopes and changes in TMS-evoked potentials, will serve as a predictor of both acute mood response and risk of relapse. Integrating EEG and TMS-EEG metrics with BDNF trajectories provides a multimodal framework for characterizing the antidepressant mechanisms underlying chronotherapy. This is consistent with ENIGMA and EEG literature showing modest but consistent alterations in frontal–limbic circuits and the balance between excitation and inhibition in patients with remitted affective disorders.

## Perspectives

**Fig. 1:**
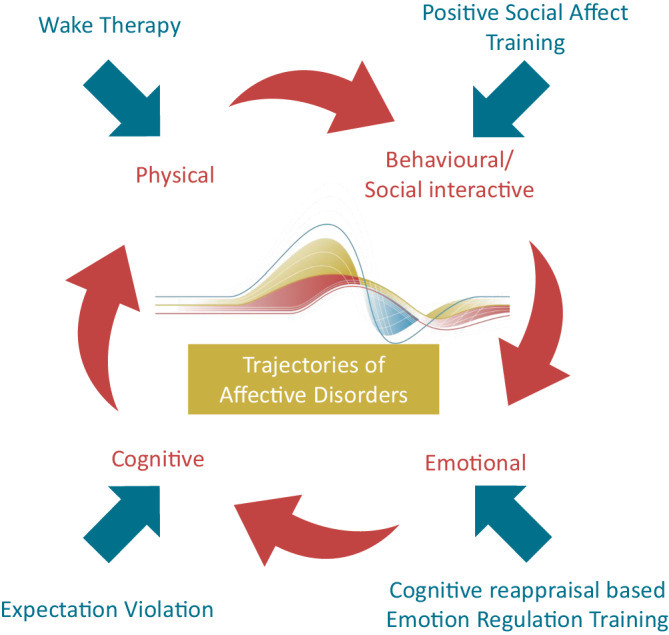
Overview of mechanism-targeted interventions to treat affective disorders. Note that the assignment to one of the four mechanisms is a simplification, as in reality the mechanisms overlap

By investigating these four intervention mechanisms, we address key components involved in the development and recurrence of affective disorders (see Fig. 1).

These four key mechanisms are derived from the literature on MDD [13]. However, they are not specific to depression but are transdiagnostic in nature, aligning with the concept of transdiagnostic dimensional constructs, such as research domain criteria [41, 42], which underlie mental disorders. Further, our interventions are unlikely to affect only targeted mechanisms, but may also influence other mechanisms directly or indirectly. While this may be considered a limitation from an experimental perspective—where controlling for potential confounders is crucial for drawing causal conclusions—the clinical perspective emphasizes that mechanisms are embedded within networks. Interventions may affect specific nodes in these networks, thereby altering the broader network structure [43]. Furthermore, we will include this as part of our investigations and will assess for each participant the potential intervention effects on the other non-targeted mechanisms. This will enable us to also investigate the interaction between different mechanisms. Interestingly, the National Institute of Mental Health (NIMH, [44]) recently emphasized the importance of investigating target mechanisms that influence disease trajectories.

With the focus on mechanisms, we are not alone. Several mechanism-based approaches have been shown to be effective in treating MDD (among others, cognitive behavioral analysis system of psychotherapy [CBASP], [45] and behavioral activation, [46]) and BD (among others, acceptance and commitment therapy [ACT], [47]). Unfortunately, to date, this has not sufficiently reduced the high recurrence rates and treatment non-responses (yet). Our approach complements the existing important work on the individualization and optimization of psychotherapeutic interventions by conducting a large-scale longitudinal study investigating the mechanisms (in animals and humans, [9]), their potential interactions, and their modifiability within a single cohort and across multiple levels [9–12]. This holds promise for improving our understanding of how to positively influence the trajectories of affective disorders, as well as for making valid predictions about which interventions work best for whom and why.

### Limitations

Nevertheless, it is important to anticipate certain limitations of our approach. Firstly, as noted above, the mechanisms targeted by the intervention are non-selective. Secondly, due to the limitations of the current funding period, we are investigating the intervention in distinct subsamples of GEMCO. This prevents us from comparing the effects of each intervention in the same patient, as would be possible in a modular treatment approach realized in an adaptive trial design. Thirdly, we do not (yet) expect to observe clinically significant changes but rather to test the malleability of mechanisms in response to the intervention. Thus, subsequent optimization studies will likely be required before clinical implementation.

In summary, we are testing the modifiability of these mechanisms in parallel and longitudinally, which has not been done before. This approach is promising because we might be able to (1) demonstrate in proof-of-concept studies that our interventions are suitable for targeting one of the four mechanisms, (2) thereby influencing clinical trajectories and (3) obtaining initial evidence on the circumstances or patient subgroups in which specific interventions might be particularly promising, thus paving the way for the evidence-based development of innovative personalized treatment options.

## Practical conclusion

- The treatment of affective disorders may benefit from longitudinal investigation of the modifiability of key cognitive–emotional mechanisms such as *emotion regulation, expectation, social cognition, *and *circadian rhythms*.
- Interventions aimed at enhancing modifiability within these mechanisms could have potential to positively influence disease trajectories (e.g., faster remission rates, reduced relapses, decreased symptom severity).
- Regarding care, mechanism-adapted interventions could enrich existing foci of therapeutic treatment and improve response rates.
- Specifically, interventions such as *cognitive reappraisal-based emotion regulation training, expectancy violation, positive social affect training,* and *wake therapy *could—with ongoing research—be adapted into therapeutic practice.
- These interventions could potentially serve as one module in a modular treatment approach.
- Prospectively, evidence regarding the most suitable application context and target group of these interventions could facilitate the development of personalized treatment approaches.

## Supplementary Information

Table e1: Interventions and key variables.

## Declarations

For this article no studies with human participants or animals were performed by any of the authors. All studies mentioned were in accordance with the ethical standards indicated in each case.

## References

[1] BÄK, KBV, AWMF (2022) Nationale Versorgungsleitlinie Unipolare Depression – Langfassung, Version 3.2. www.leitlinien.de/depression. Accessed 29 May 2025

[2] DGBS e. V., DGPPN e. V. (2019) S3-Leitlinie zur Diagnostik und Therapie Bipolarer Störungen. Langversion, 2019

[3] Wiegand HF, Sievers C, Schillinger M, Godemann F (2016) Major depression treatment in Germany-descriptive analysis of health insurance fund routine data and assessment of guideline-adherence. J Affect Disord 189:246–253 26454184 10.1016/j.jad.2015.09.013

[4] Fernández A, Haro JM, Martinez-Alonso M, Demyttenaere K, Brugha TS, Autonell J et al (2007) Treatment adequacy for anxiety and depressive disorders in six European countries. Br J Psychiatry 190(2):172–173 17267936 10.1192/bjp.bp.106.023507

[5] Cuijpers P, Miguel C, Ciharova M, Harrer M, Basic D, Cristea IA et al (2024) Absolute and relative outcomes of psychotherapies for eight mental disorders: a systematic review and meta-analysis. World Psychiatry 23(2):267–275 38727072 10.1002/wps.21203 PMC11083862

[6] Hardeveld F, Spijker J, De Graaf R, Nolen WA, Beekman ATF (2010) Prevalence and predictors of recurrence of major depressive disorder in the adult population. Acta Psychiatr Scand 122(3):184–191 20003092 10.1111/j.1600-0447.2009.01519.x

[7] Lam RW, Yatham LN, McGirr A, Gignac A (2015) Recovery and recurrence following a first episode of mania: a systematic review and meta-analysis of prospectively characterized cohorts. J Clin Psychiatry 76(9):1241–1248 25845021 10.4088/JCP.14r09245

[8] Kessing LV, Andersen PK, Vinberg M (2018) Risk of recurrence after a single manic or mixed episode—a systematic review and meta-analysis. Bipolar Disord 20(1):9–17 29239075 10.1111/bdi.12593

[9] Kanske P, Alexander N, Bernhardt N, Ehrlich S, Groß J, Culmsee C, Leehr EJ, Jansen A, Jüngling K, Ritter P, Straube B, Wessing I, Kircher T, Wöhr M (2025) Key mechanisms of affective disorders. Der Nervenarzt. 10.1007/s00115-025-01920-9 10.1007/s00115-025-01920-9 PMC12953480 41212184

[10] Kircher T, Alexander N, Bauer M, Dannlowski U, Ebner-Priemer UW, Kanske P et al (2025) The SFB/TRR 393 Collaborative Research Centre: trajectories of affective disorders. Nervenarzt. 10.1007/s00115-025-01886-8 40940529 10.1007/s00115-025-01886-8 PMC12953339

[11] Dannlowski U, Alexander N, Ebner-Priemer UW Trajectories of Affective Disorders—the central structures of the CRC/TRR 393. Nervenarzt (in preparation) 10.1007/s00115-025-01921-8 PMC12953259 41296286

[12] Ebner-Priemer UW, Alferink J, Bauer M Trajectories of affective disorders: neurobiological mechanisms during symptom change. Nervenarzt 97: (in preparation) 10.1007/s00115-025-01917-4 PMC12953256 41263959

[13] LeMoult J, Gotlib IH (2019) Depression: a cognitive perspective. Clin Psychol Rev 69:51–66 29961601 10.1016/j.cpr.2018.06.008 PMC11884012

[14] Tamir M, Ford BQ (2012) When feeling bad is expected to be good: emotion regulation and outcome expectancies in social conflicts. Emotion 12(4):807–816 21728413 10.1037/a0024443

[15] Antúnez JM (2020) Circadian typology is related to emotion regulation, metacognitive beliefs and assertiveness in healthy adults. PLoS ONE 15(3):e230169 32168366 10.1371/journal.pone.0230169 PMC7069650

[16] Salazar Kämpf M, Adam L, Rohr MK, Exner C, Wieck C (2023) A meta-analysis of the relationship between emotion regulation and social affect and cognition. Clin Psychol Sci 11(6):1159–1189

[17] Förster K, Kurtz M, Konrad A, Kanske P (2022) Emotional reactivity, emotion regulation, and social emotions in affective disorders. Z Klin Psychol Psychother. 10.1026/1616-3443/a000648

[18] Aldao A, Sheppes G, Gross JJ (2015) Emotion regulation flexibility. Cogn Ther Res 39(3):263–278

[19] Hofmann SG, Sawyer AT, Fang A, Asnaani A (2012) Emotion dysregulation model of mood and anxiety disorders. Depress Anxiety 29(5):409–416 22430982 10.1002/da.21888

[20] Cohen N, Ochsner KN (2018) From surviving to thriving in the face of threats: the emerging science of emotion regulation training. Curr Opin Behav Sci 24:143 31187051 10.1016/j.cobeha.2018.08.007 PMC6559737

[21] Dannlowski U, Pfennig A, Ebner-Priemer UW, Falkenberg I, Hahn T, Hamidreza J et al (2026) Trajectories of Affective Disorders—the central structures of the CRC/TRR 393 Irina Falkenberg, Tim Hahn, Hamidreza Jamalabadi, Andreas Jansen, Tilo Kircher, Ralph Müller-Pfefferkorn, Andrea Pfennig, Michael Smolka, Frederike Stein, Benjamin Straube. Nervenarzt 97: (in preparation) 10.1007/s00115-025-01921-8 PMC12953259 41296286

[22] Rief W, Joormann J (2019) Revisiting the cognitive model of depression: the role of expectations. Clin Psychol Eur 1(1):1–19

[23] Rief W, Sperl MFJ, Braun-Koch K, Khosrowtaj Z, Kirchner L, Schäfer L et al (2022) Using expectation violation models to improve the outcome of psychological treatments. Clin Psychol Rev 98:102212 36371900 10.1016/j.cpr.2022.102212

[24] Redlich R, Dohm K, Grotegerd D, Opel N, Zwitserlood P, Heindel W et al (2015) Reward processing in unipolar and bipolar depression: a functional MRI study. Neuropsychopharmacology 40(11):2623–2631 25881114 10.1038/npp.2015.110 PMC4569953

[25] Potsch L, Rief W (2024) How to improve reward sensitivity—Predictors of long-term effects of a randomized controlled online intervention trial. J Affect Disord 367:647–657 39243822 10.1016/j.jad.2024.09.007

[26] Ewen ACI, Bleichhardt G, Rief W, Von Blanckenburg P, Wambach K, Wilhelm M (2023) Expectation focused and frequency enhanced cognitive behavioural therapy for patients with major depression (EFFECT): a study protocol of a randomised active-control trial. BMJ Open 13(3):e65946 36948546 10.1136/bmjopen-2022-065946 PMC10040046

[27] Potsch L, Rief W (2023) Transdiagnostic considerations of the relationship between reward sensitivity and psychopathological symptoms—a cross-lagged panel analysis. BMC Psychiatry 23(1):650 37667190 10.1186/s12888-023-05139-3 PMC10478275

[28] Hamilton, M (1960) A Rating Scale for depression. Journal of Neurological Neurosurgery 23(56):56–63 10.1136/jnnp.23.1.56 PMC495331 14399272

[29] Hames JL, Hagan CR, Joiner TE (2013) Interpersonal processes in depression. Annu Rev Clin Psychol 9(1):355–377 23297787 10.1146/annurev-clinpsy-050212-185553

[30] Engert V, Linz R, Grant JA (2019) Embodied stress: the physiological resonance of psychosocial stress. Psychoneuroendocrinology 105:138–146 30594324 10.1016/j.psyneuen.2018.12.221

[31] Trautwein FM, Kanske P, Böckler A, Singer T (2017) Differential benefits of mental training types for attention, compassion, and theory of mind. Cognition 194:104039 10.1016/j.cognition.2019.104039 PMC6891878 31450018

[32] Kok BE, Coffey KA, Cohn MA, Catalino LI, Vacharkulksemsuk T, Algoe SB et al (2013) How positive emotions build physical health: perceived positive social connections account for the upward spiral between positive emotions and vagal tone. Psychol Sci 24(7):1123–1132 23649562 10.1177/0956797612470827

[33] Hildebrandt LK, McCall C, Singer T (2017) Differential effects of attention-, compassion-, and socio-cognitively based mental practices on self-reports of mindfulness and compassion. Mindfulness 8(6):1488–1512 29201246 10.1007/s12671-017-0716-z PMC5693975

[34] Kok BE, Singer T (2017) Phenomenological fingerprints of four meditations: differential state changes in affect, mind-wandering, meta-cognition, and Interoception before and after daily practice across 9 months of training. Mindfulness 8(1):218–231 28163798 10.1007/s12671-016-0594-9 PMC5241345

[35] Konrad AC, Engert V, Albrecht R, Dobel C, Döring N, Haueisen J et al (2023) A multicenter feasibility study on implementing a brief mindful breathing exercise into regular university courses. Sci Rep 13(1):7908–7916 37193767 10.1038/s41598-023-34737-0 PMC10186318

[36] Förster K, Kanske P (2022) Upregulating positive affect through compassion: psychological and physiological evidence. Int J Psychophysiol 176:100–107 35358613 10.1016/j.ijpsycho.2022.03.009

[37] Mekelburg A, Maliske L, Kirby J, Kanske P, Förster K (2025) Functional neural plasticity after compassion-based interventions: a scoping review of longitudinal neuroimaging studies. J Affect Disord (https://www.sciencedirect.com/science/article/pii/S0165032725007633) 10.1016/j.jad.2025.05.006 40334852

[38] Liebmann C, Konrad AC, Singer T, Kanske P (2023) Differential reduction of psychological distress by three different types of meditation-based mental training programs: a randomized clinical trial. Int J Clin Health Psychol 23(4):100388 37214346 10.1016/j.ijchp.2023.100388 PMC10199252

[39] Gottlieb JF, Benedetti F, Geoffroy PA, Henriksen TEG, Lam RW, Murray G et al (2019) The chronotherapeutic treatment of bipolar disorders: a systematic review and practice recommendations from the ISBD task force on chronotherapy and chronobiology. Bipolar Disord 21(8):741–773 31609530 10.1111/bdi.12847

[40] Zangani C, Casetta C, Saunders AS, Donati F, Maggioni E, D’Agostino A (2020) Sleep abnormalities across different clinical stages of bipolar disorder: a review of EEG studies. Neurosci Biobehav Rev 118:247–257 32738263 10.1016/j.neubiorev.2020.07.031

[41] Insel T, Cuthbert B, Garvey M, Heinssen R, Pine DS, Quinn K et al (2010) Research domain criteria (RdoC): toward a new classification framework for research on mental disorders. Am J Psychiatry 167(7):748–751 20595427 10.1176/appi.ajp.2010.09091379

[42] Insel TR (2014) The NIMH research domain criteria (RdoC) project. Precis Med Psychiatry 171(4):395–397. 10.1176/appi.ajp.2014.14020138 10.1176/appi.ajp.2014.14020138 24687194

[43] Hofmann SG (2025) A network control theory of dynamic systems approach to personalize therapy. Behav Ther 56(1):199–212 39814513 10.1016/j.beth.2024.10.006

[44] Zucker NL, Strauss GP, Smyth JM, Scherf KS, Brotman MA, Boyd RC et al (2025) Experimental therapeutics: opportunities and challenges stemming from the national institute of mental health workshop on novel target discovery and psychosocial intervention development. Perspect Psychol Sci 20(3):485–502 37874961 10.1177/17456916231197980 PMC11039571

[45] Negt P, Brakemeier EL, Michalak J, Winter L, Bleich S, Kahl KG (2016) The treatment of chronic depression with cognitive behavioral analysis system of psychotherapy: a systematic review and meta-analysis of randomized-controlled clinical trials. Brain Behav 6(8):e486 27247856 10.1002/brb3.486 PMC4864084

[46] Cuijpers P, Karyotaki E, Harrer M, Stikkelbroek Y (2023) Individual behavioral activation in the treatment of depression: a meta analysis. Psychother Res 33(7):886–897 37068380 10.1080/10503307.2023.2197630

[47] El-Sayed MM, Elhay ESA, Taha SM, Khedr MA, Mansour FSA, El-Ashry AM (2023) Efficacy of acceptance and commitment therapy on impulsivity and suicidality among clients with bipolar disorders: a randomized control trial. BMC Nurs 22:271 37592290 10.1186/s12912-023-01443-1 PMC10433608

