# Supplementary material for: What to target? Interventions to modulate key mechanisms underlying the trajectories of affective disorders in the transregional Collaborative Research Center 393
Source: Nervenarzt. 2025 Nov 27;97(2):154–60. doi: 10.1007/s00115-025-01929-0 (PMC12953325; doi:10.1007/s00115-025-01929-0)
Supplement: Supplementary file 1 — Table e1: Interventions and key variables. [file 115_2025_1929_MOESM1_ESM.pdf]

Table e1. Interventions and key variables.

Mechanism targeted	Intervention	Form of intervention	Duration	Sample	Control Condition	Primary outcome
emotion regulation	cognitive reappraisal-based emotion regulation training	app-based	8-week	patients with current or remitted MDD	cognitive exercises (attention, verbal, executive functioning, and spatial perception)	change in ER flexibility (ER choice sensitivity and variability from baseline to follow-up); MRI-based structural plasticity (fronto-limbic brain regions) and functional brain plasticity in ER and emotion perception networks between baseline and follow-up
expectation	expectation-focused treatment	face-to-face group setting	5 weeks, twice a week (10 sessions)	patients with an acute major depressive episode	1. reward sensitivity-focused treatment (active control)<br>2. waiting list control (passive control)	Change in HAMD and MRI-based parameters derived from mechanism-specific paradigms (fMRI), with insula ROIs and dlPFC changes in task-based activation post- vs. pre-treatment
social cognition	positive affect training with partner	online setting with app support	8 weeks	patients with MDD currently euthymic	non-social mindfulness-based intervention	changes (pre- to post-intervention) in self-reported empathy assessed during the EmpaTom – an experimental task to assess empathy
circadian rhythms	wake therapy	Face-to-face	4 days	patients with current episode of depression (bipolar or unipolar)	no control condition	modulation of synaptic plasticity in the left dlPFC quantified with TMS-evoked EEG responses and complemented by resting-state EEG aperiodic slope and theta power

Note. dlPFC= dorsolateral prefrontal cortex. EEG= electroencephalography. ER= emotion regulation. fMRI= functional magnetic resonance imaging. MDD= Major Depressive Disorder. TMS=transcranial magnetic stimulation
